# Prediction of additive, epistatic, and dominance effects using models accounting for incomplete inbreeding in parental lines of hybrid rye and sugar beet

**DOI:** 10.3389/fpls.2023.1193433

**Published:** 2023-11-02

**Authors:** Peter Skov Kristensen, Pernille Sarup, Dario Fé, Jihad Orabi, Per Snell, Linda Ripa, Marius Mohlfeld, Thinh Tuan Chu, Joakim Herrström, Ahmed Jahoor, Just Jensen

**Affiliations:** ^1^ Center for Quantitative Genetics and Genomics, Aarhus University, Aarhus, Denmark; ^2^ Research and Development, Nordic Seed A/S, Odder, Denmark; ^3^ Research Division, DLF Seeds A/S, Store Heddinge, Denmark; ^4^ Research and Development, DLF Beet Seed AB, Landskrona, Sweden; ^5^ Breeding, Nordic Seed Germany GmbH, Nienstädt, Germany; ^6^ Department of Plant Breeding, Swedish University of Agricultural Sciences, Alnarp, Sweden

**Keywords:** genomic selection, non-additive genetic effects, Gca and sca, hybrid breeding, heterotic groups, inbreeding, grain yield, root yield

## Abstract

Genomic models for prediction of additive and non-additive effects within and across different heterotic groups are lacking for breeding of hybrid crops. In this study, genomic prediction models accounting for incomplete inbreeding in parental lines from two different heterotic groups were developed and evaluated. The models can be used for prediction of general combining ability (GCA) of parental lines from each heterotic group as well as specific combining ability (SCA) of all realized and potential crosses. Here, GCA was estimated as the sum of additive genetic effects and within-group epistasis due to high degree of inbreeding in parental lines. SCA was estimated as the sum of across-group epistasis and dominance effects. Three models were compared. In model 1, it was assumed that each hybrid was produced from two completely inbred parental lines. Model 1 was extended to include three-way hybrids from parental lines with arbitrary levels of inbreeding: In model 2, parents of the three-way hybrids could have any levels of inbreeding, while the grandparents of the maternal parent were assumed completely inbred. In model 3, all parental components could have any levels of inbreeding. Data from commercial breeding programs for hybrid rye and sugar beet was used to evaluate the models. The traits grain yield and root yield were analyzed for rye and sugar beet, respectively. Additive genetic variances were larger than epistatic and dominance variances. The models’ predictive abilities for total genetic value, for GCA of each parental line and for SCA were evaluated based on different cross-validation strategies. Predictive abilities were highest for total genetic values and lowest for SCA. Predictive abilities for SCA and for GCA of maternal lines were higher for model 2 and model 3 than for model 1. The implementation of the genomic prediction models in hybrid breeding programs can potentially lead to increased genetic gain in two different ways: I) by facilitating the selection of crossing parents with high GCA within heterotic groups and II) by prediction of SCA of all realized and potential combinations of parental lines to produce hybrids with high total genetic values.

## 1 Introduction

Hybrid varieties of important crops, such as maize (*Zea mays* L.), rye (*Secale cereale* L.), and sugar beet (*Beta vulgaris* L. ssp. *vulgaris*), are widely cultivated and perform considerably better than inbred or population varieties (Campbell, 1990; Duvick, 2005; Laidig et al., 2017). The improved performance is due to heterosis or hybrid vigor, which occurs when genetically different lines are crossed (Labroo et al., 2021). However, breeding programs for hybrid crops typically require many resources and have long breeding cycles (Longin et al., 2012). Both of these factors limit genetic gain in hybrid crops compared to line breeding programs. Rye and sugar beet are both crops that are commonly cultivated as hybrids, which are produced by crossing inbred lines from different heterotic groups. In the current study, data from three-way hybrids and their parental components were evaluated. First, two-way crosses between cytoplasmic male-sterile (MS) and non-restorer (NR) lines from one heterotic group are produced, and the resulting male-sterile offspring are then crossed with a pollinator or restorer (R) line from a different heterotic group to produce the three-way hybrids. MS lines do not produce viable pollen. When MS lines are crossed with an NR line, the offspring remain male-sterile, and when MS lines are crossed with a restorer line, the offspring become male-fertile and can produce viable pollen (Vendelbo et al., 2020).

Many studies have shown that genomic prediction can be applied to a broad range of complex traits in animals and crops to increase rate of genetic gain and improve effectiveness in breeding programs (Meuwissen et al., 2016; Crossa et al., 2017; Kristensen et al., 2019). For genomic prediction, numerous of genome-wide markers are used to predict genomic breeding values of lines, based on a training set consisting of lines that have both been genotyped and phenotyped (Meuwissen et al., 2001). Many important agronomic traits have a complex genetic architecture, i.e. they are controlled by many QTL, each having a small effect (Würschum et al., 2011; Hackauf et al., 2017). For such traits, genomic prediction models have been shown to be more accurate than marker-assisted selection based on few markers (Wang et al., 2014; Arruda et al., 2016).

For optimal use of genomic selection in breeding programs for hybrid crops, it is essential to have models that can predict both genomic breeding values of lines within heterotic groups as well as the total genetic value of the hybrids (Technow et al., 2012). However, studies of genomic prediction in hybrid rye and sugar beet have so far been conducted using datasets and models, where it was not possible to separately estimate additive genetic effects of each parent and non-additive genetic effects of the hybrids, which limits the practical use for breeding (Hofheinz et al., 2012; Würschum et al., 2013; Wang et al., 2014; Auinger et al., 2016; Schulthess et al., 2016; Bernal-Vasquez et al., 2017). Non-additive genetic effects consist of dominance and epistatic deviations from the additive allele substitution effects. Dominance deviations are due to interactions between different alleles within a locus. Epistatic deviations are due to interactions between alleles across loci, and can consist of additive by additive, additive by dominance, dominance by dominance, and any higher order interactions (Falconer and Mackay, 1996).

González-Diéguez et al. (2021) developed a “GCA-model” to predict the performance of hybrids made by crossing completely inbred parental lines from two different heterotic groups. In this model, hybrid performance could be split into additive effects from the parental lines in each heterotic group, epistatic deviations both within and across the two groups, and dominance deviations. Genetic effects might differ between heterotic groups due to differences in allele frequencies, differences in linkage disequilibrium between QTL and markers, markers linked to opposite phases of the QTL, or due to QTL that segregate in one group, but not in the other (Vendelbo et al., 2020; Vendelbo et al., 2021a). Therefore, effects were defined separately for each of the two heterotic groups in the model (González-Diéguez et al., 2021). If parental lines are completely inbred, general combining ability (GCA) can be estimated as the sum of the additive and within-line epistatic deviations for each parent, while specific combining ability (SCA) can be estimated as the sum of the dominance and across-group epistatic deviations. If parental lines are not inbred, within-line epistatic deviations are not always transmitted to the offspring due to recombination during meiosis, and GCA is therefore estimated based on additive genetic effects only. González-Diéguez et al. (2021) evaluated their model using data from a hybrid maize breeding program, and the GCA-model resulted in high prediction accuracies for grain yield (0.80 to 0.92 based on different cross-validation strategies).

In the current study, the GCA-model was extended from predicting the performance of two-way hybrids produced from fully inbred lines to predicting the performance of three-way hybrids produced from parental lines with arbitrary levels of inbreeding. The models were evaluated using data from two commercial breeding programs for hybrid rye and sugar beet. The genetic variation and the degree of homozygosity in the parental lines of the different heterotic groups of the breeding programs were investigated. Rye grain yield and sugar beet root yield were analyzed, and different cross-validation strategies were used for evaluating the predictive abilities. The models can be used in breeding programs to predict SCA for all realized and potential hybrids as well as GCA for all genotyped individuals in the two parental heterotic groups.

## 2 Materials and methods

### 2.1 Phenotypic and genotypic data

Data from the rye breeding program of the company Nordic Seed and from the sugar beet breeding program of the company DLF Beet Seed was used (**Supplementary Tables**). The phenotypic data consisted of yield of three-way hybrids tested in replicated multi-location trials across several European countries from 2016-2022 for rye and from 2012-2022 for sugar beet. Environmental effects and spatial variations in the fields were accounted for by the fixed effects in the genomic models described below. For rye, grain yield of plots was corrected to a moisture content of 15%. Replications within a trial were either treated with fungicides and growth regulators or were untreated. For sugar beet, root yield of plots was recorded as fresh weight, and all replicates were treated with fungicides. The three-way hybrids were produced by first crossing an MS and an NR line. The MS lines were derived from NR lines via several generations of backcrossing. Thus, the MS and NR both belong to the same heterotic group. The two-way hybrid from the cross between MS and NR was then crossed with an R line from a second heterotic group to produce a three-way hybrid. The number of tested three-way hybrids and of the parental components used for producing the hybrids are shown in **Table 1**. Parental components of the hybrids (MS, NR, and R lines) were genotyped with SNP chip arrays. For rye lines, DNA was extracted from leaves of seedlings, and genotyping was carried out by TraitGenetics GmbH (Germany) using a custom Illumina Infinium 15K wheat + 5K rye SNP iSelect ultra HD chip array (Vendelbo et al., 2020). For sugar beet lines, DNA was extracted from first true leaves using the sbeadex™ Magnetic Bead Kit (LGC) in accordance with the manufacturer’s instructions, and genotyping was carried out by Eurofins Genomics Europe (Denmark) using a custom 21K Sugar beet Affymetrix Axiom microarray. After filtering for minor allele frequency and missing values (thresholds of 0.1% and 20%, respectively), 5,768 SNPs were included in the analyses for rye and 6,514 SNPs for sugar beet.

**Table 1:** Number of three-way hybrids, parental components, and plots of three-way hybrids.

Crop	No. of components		No. of plots
Rye	MS lines	13	
	NR lines	11	
	R lines	302	
	Two-way crosses	33	
	Three-way hybrids	570	12,326
Sugar beet	MS lines	17	
	NR lines	58	
	R lines	223	
	Two-way crosses	83	
	Three-way hybrids	657	47,703

### 2.2 Genomic prediction models

Three genomic prediction models were evaluated. Model 1 (M1) was based on the GCA-model developed by González-Diéguez et al. (2021). Here, it is assumed that each hybrid was produced from completely inbred parental lines. The maternal lines belonged to heterotic group 1, and the paternal lines belonged to heterotic group 2. Genotypes of the two-way crosses were imputed from the genotypes of the MS and NR lines, and these genotypes were used in the calculation of genomic relationship matrices. If a SNP marker was heterozygous in a parental line (alleles *B_1_b_1_* in group 1 or *B_2_b_2_* in group 2), it was randomly assigned to one of the two homozygous genotypes (*B_1_B_1_*/*b_1_b_1_* or *B_2_B_2_/b_2_b_2_*, respectively).

In model 2 (M2) and model 3 (M3), the genotypes of the MS and NR lines were used directly in the calculations in order to better utilize the genomic relationship between the lines within group 1. In M2, the R lines and the two-way crosses could have arbitrary levels of inbreeding, while the MS and NR lines were assumed completely inbred. Any heterozygous SNPs in the MS and NR lines were randomly assigned to one of the two homozygous genotypes. In M3, the R lines, the two-way crosses, and the MS and NR lines could all have arbitrary levels of inbreeding. If all parental components are completely inbred, then M2 and M3 are equivalent to M1.

Thus, the M1 model was:


(1) $$\begin{array}{l} y=Xb+T_{1}{ɡ}_{A_{\left(1\right)}}+T_{2}{ɡ}_{A_{\left(2\right)}}+T_{3}{ɡ}_{D}+T_{1}{ɡ}_{AA_{\left(1\right)}}+T_{2}{ɡ}_{AA_{\left(2\right)}}+T_{3}{ɡ}_{AA_{\left(3\right)}}\\ \;+T_{1}r_{\left(1\right)}+T_{2}r_{\left(2\right)}+T_{3}r_{\left(3\right)}+T_{4}k+T_{5}l+T_{6}m+e \end{array}$$

where ***y*** is the vector of phenotypes of the hybrids; ***X*** is the design matrix for fixed effects (year x location x trial. For rye, treatment was included as a second fixed effect); ***b*** is the vector of fixed effects; ***T***_1_ and ***T***_2_ are design matrices to assign hybrids to their parental lines in heterotic group 1 and 2, ${ɡ}_{A_{\left(1\right)}}$ and ${ɡ}_{A_{\left(2\right)}}$ are vectors of additive genetic effects from parental lines from group 1 and 2, respectively, with ${ɡ}_{A_{\left(1\right)}}∼N(0,G_{A_{\left(1\right)}}\sigma_{A_{\left(1\right)}}^{2})$ and ${ɡ}_{A_{\left(2\right)}}∼N(0,G_{A_{\left(2\right)}}\sigma_{A_{\left(2\right)}}^{2})$ , where $\sigma_{A_{\left(1\right)}}^{2}$ and $\sigma_{A_{\left(2\right)}}^{2}$ are additive genetic variances and $G_{A_{\left(1\right)}}$ and $G_{A_{\left(2\right)}}$ are additive genomic relationship matrices; ${ɡ}_{AA_{\left(1\right)}}$ and ${ɡ}_{AA_{\left(2\right)}}$ are vectors of additive-by-additive epistatic effects within heterotic group 1 and 2, respectively, with ${ɡ}_{AA_{\left(1\right)}}∼N(0,G_{AA_{\left(1\right)}}\sigma_{AA_{\left(1\right)}}^{2})$ and $G_{AA_{\left(2\right)}}∼N(0,G_{AA_{\left(2\right)}}\sigma_{AA_{\left(2\right)}}^{2})$ , where $\sigma_{AA_{\left(1\right)}}^{2}$ and $\sigma_{AA_{\left(2\right)}}^{2}$ are epistatic genetic variances within each heterotic group and $G_{AA_{\left(1\right)}}$ and $G_{AA_{\left(2\right)}}$ are within-group epistatic genomic relationship matrices; ***T*** *_3_* is the design matrix for the effects of the hybrids; ${ɡ}_{AA_{\left(3\right)}}$ is the vector of additive-by-additive epistatic effects between alleles from heterotic group 1 and 2, respectively, with ${ɡ}_{AA_{\left(3\right)}}∼N(0,G_{AA_{\left(3\right)}}\sigma_{AA_{\left(3\right)}}^{2})$ , where $\sigma_{AA_{\left(3\right)}}^{2}$ is epistatic genetic variance between the heterotic groups and $G_{AA_{\left(3\right)}}$ is the across-group epistatic genomic relationship matrix; ***g*** *_D_* is the vector of genetic dominance deviations due to within locus interactions between alleles from different heterotic groups with ${ɡ}_{D}∼\;N\left(0,D\sigma_{D}^{2}\right)$ , where $\sigma_{D}^{2}$ is genetic dominance variance and ***D*** is the dominance relationship matrix across hybrids; ***r*_(1)_**, ***r***_(2)_ and ***r*_(_**_3_ **_)_** are vectors of residual genetic effects of lines from group 1 and 2 and of the hybrids, respectively, with $r_{\left(1\right)}∼\;N\left(0,I_{r_{\left(1\right)}}\sigma_{r_{\left(1\right)}}^{2}\right)$ , $r_{\left(2\right)}∼\;N\left(0,I_{r_{\left(2\right)}}\sigma_{r_{\left(2\right)}}^{2}\right)$ and $r_{\left(3\right)}∼\;N\left(0,I_{r_{\left(3\right)}}\sigma_{r_{\left(3\right)}}^{2}\right)$ , where $I_{r_{\left(1\right)}}$ , $I_{r_{\left(2\right)}}$ , and $I_{r_{\left(3\right)}}$ are identity matrices and $\sigma_{r_{\left(1\right)}}^{2}$ , $\sigma_{r_{\left(2\right)}}^{2}$ and $\sigma_{r_{\left(3\right)}}^{2}\;$ are residual genetic variances; ***T_4_***, ***T_5_***, and ***T_6_*** are design matrices for random effects of interactions between year x location and maternal parent, paternal parent or treatment (only included for rye), respectively, and ***k***, ***l***, and ***m*** are the vectors of the random effects of the interactions with $k\;∼\;N\left(0,I_{k}\sigma_{k}^{2}\right)$ , $l\;∼\;N\left(0,I_{l}\sigma_{l}^{2}\right)$ , and $m\;∼\;N\left(0,I_{m}\sigma_{m}^{2}\right)$ , where ***I_k_***, ***I_l_***, and ***I_m_*** are identity matrices and $\sigma_{k}^{2}$ , $\sigma_{l}^{2}$ , and $\sigma_{m}^{2}$ , are variances for the interactions; ***e*** is the vector of random residual effects with $e\;∼\;N\left(0,I_{e}\sigma_{e}^{2}\right)$ , where ***I_e_*** is an identity matrix and $\sigma_{e}^{2}$ is residual variance.

For M1, genomic relationship matrices were calculated as proposed by González-Diéguez et al. (2021):

Additive genomic relationship matrix for heterotic group 1:


(2) $$G_{A_{\left(1\right)}}=\;\frac{Z_{1}Z_{1}^{'}}{\sum_{i}^{nsnp}p_{1_{i}}q_{1_{i}}}$$

where $p_{1_{i}}$ and $q_{1_{i}}$ are the frequencies of allele $B_{1_{i}}$ and $b_{1_{i}}$ for the *i^th^* marker, respectively, and ***Z*** *_1_* = ***M***_1_ - ***P***_1_; ***M***_1_ is a matrix with genotypes of parental lines in group 1 coded as 0 for genotype *b_1_b_1_* and 1 for genotype *B_1_B_1_* for each marker; ***P***_1_ is a matrix where each column contains the allele frequencies of $B_{1}$ , and *nsnp* is number of markers.

Additive-by-additive epistatic relationship matrix for lines within group 1 was calculated as the Hadamard product of the additive genomic relationship matrix for group 1 scaled by the trace of the resulting matrix divided by the number of lines in group 1 to get an average diagonal of 1:


(3) $$G_{AA_{\left(1\right)}}=\frac{G_{A_{\left(1\right)}}⨀\;G_{A_{\left(1\right)}}}{tr\left(G_{A_{\left(1\right)}}⨀\;G_{A_{\left(1\right)}}\right)/n_{1}}$$

The additive and epistatic genomic relationship matrices for heterotic group 2 were calculated in same way as for group 1.

Additive-by-additive epistatic relationship matrix between lines in group 1 and 2:


(4) $$G_{AA_{\left(3\right)}}=\frac{T_{1}G_{A_{\left(1\right)}}T_{1}^{'}⨀\;T_{2}G_{A_{\left(2\right)}}T_{2}^{'}}{tr\left(T_{1}G_{A_{\left(1\right)}}T_{1}^{'}⨀\;T_{2}G_{A_{\left(2\right)}}T_{2}^{'}\right)/n_{H}}$$

where *n_H_* is the number of hybrids. The matrices $G_{AA_{\left(3\right)}}$ and ***D*** can both include realized hybrids as well as all potential crosses of the parental lines, so the crosses with the largest effects can be predicted even though they are not yet phenotypically tested.

Dominance relationship matrix of dominance interactions between alleles from different heterotic groups:


(5) $$D=\;\frac{W_{1}W_{1}^{'}}{\sum_{i}^{nsnp}\left(4p_{1_{i}}q_{1_{i}}p_{2_{i}}q_{2_{i}}\right)}$$

where $p_{1_{i}}$ , $q_{1_{i}}$ , $p_{2_{i}}$ and $q_{2_{i}}$ are the frequencies of the alleles $B_{1_{i}}$ and $b_{1_{i}}$ in heterotic group 1 and $B_{2_{i}}$ and $b_{2_{i}}$ in heterotic group 2 for the *i^th^* marker, respectively, and ***W_1_*** is a matrix with a row for each hybrid and a column for each marker (González-Diéguez et al., 2021). The elements of ***W_1_*** are shown in **Table 2**.

**Table 2:** Elements of W_1_, W_2_, and W_3_ for each marker in the hybrids from crosses between parental lines from group 1 and group 2, which are used in the calculation of the dominance relationship matrix for M1, M2, and M3, respectively*.

Parental genotypes	w_1_	w_2_	w_3_
*(B_1_B_1_ x B_1_B_1_) x B_2_B_2_*	$-2q_{1}q_{2}$	$-2q_{1}q_{2}$	$-2q_{1}q_{2}$
*(B_1_B_1_ x B_1_B_1_) x B_2_b_2_*		$-\left(p_{1}-1\right)\left(2p_{2}-1\right)$	$-\left(p_{1}-1\right)\left(2p_{2}-1\right)$
*(B_1_B_1_ x B_1_B_1_) x b_2_b_2_*	$2q_{1}p_{2}$	$2q_{1}p_{2}$	$2q_{1}p_{2}$
*(B_1_B_1_ x B_1_b_1_) x B_2_B_2_* *(B_1_b_1_ x B_1_B_1_) x B_2_B_2_*			$\frac{-\left(4p_{1}-3\right)\left(p_{2}-1\right)}{2}$
*(B_1_B_1_ x B_1_b_1_) x B_2_b_2_* *(B_1_b_1_ x B_1_B_1_) x B_2_b_2_*			$\frac{-\left(4p_{1}-3\right)\left(2p_{2}-1\right)}{4}$
*(B_1_B_1_ x B_1_b_1_) x b_2_b_2_* *(B_1_b_1_ x B_1_B_1_) x b_2_b_2_*			$\frac{-\left(4p_{1}-3\right)p_{2}}{2}$
*(B_1_B_1_ x b_1_b_1_) x B_2_B_2_* *(B_1_b_1_ x B_1_b_1_) x B_2_B_2_* *(b_1_b_1_ x B_1_B_1_) x B_2_B_2_*		$-\left(2p_{1}-1\right)\left(p_{2}-1\right)$	$-\left(2p_{1}-1\right)\left(p_{2}-1\right)$
*(B_1_B_1_ x b_1_b_1_) x B_2_b_2_* *(B_1_b_1_ x B_1_b_1_) x B_2_b_2_* *(b_1_b_1_ x B_1_B_1_) x B_2_b_2_*		$-\frac{\left(2p_{1}-1\right)\left(2p_{2}-1\right)}{2}$	$-\frac{\left(2p_{1}-1\right)\left(2p_{2}-1\right)}{2}$
*(B_1_B_1_ x b_1_b_1_) x b_2_b_2_* *(B_1_b_1_ x B_1_b_1_) x b_2_b_2_* *(b_1_b_1_ x B_1_B_1_) x b_2_b_2_*		$-\left(2p_{1}-1\right)p_{2}$	$-\left(2p_{1}-1\right)p_{2}$
*(b_1_b_1_ x B_1_b_1_) x B_2_B_2_* *(B_1_b_1_ x b_1_b_1_) x B_2_B_2_*			$\frac{-\left(4p_{1}-1\right)\left(p_{2}-1\right)}{2}$
*(b_1_b_1_ x B_1_b_1_) x B_2_b_2_* *(B_1_b_1_ x b_1_b_1_) x B_2_b_2_*			$\frac{-\left(4p_{1}-1\right)\left(2p_{2}-1\right)}{4}$
*(b_1_b_1_ x B_1_b_1_) x b_2_b_2_* *(B_1_b_1_ x b_1_b_1_) x b_2_b_2_*			$\frac{-\left(4p_{1}-1\right)p_{2}}{2}$
*(b_1_b_1_ x b_1_b_1_) x B_2_B_2_*	$2p_{1}q_{2}$	$2p_{1}q_{2}$	$2p_{1}q_{2}$
*(b_1_b_1_ x b_1_b_1_) x B_2_b_2_*		$-p_{1}\left(2p_{2}-1\right)$	$-p_{1}\left(2p_{2}-1\right)$
*(b_1_b_1_ x b_1_b_1_) x b_2_b_2_*	$-2p_{1}p_{2}$	$-2p_{1}p_{2}$	$-2p_{1}p_{2}$

*The elements in **w_1_** are from González-Diéguez et al. (2021), and remaining elements were derived in this study (**Appendix 1**).

It should be noted that the mean heterosis of the hybrids is not estimated separately in the model but is included in the overall mean of the hybrid phenotypes. Thus, the across-group epistatic and dominance effects that are estimated are deviations of individual hybrids from the mean heterosis.

In M2, paternal R lines and maternal two-way crosses could have arbitrary levels of inbreeding, while MS and NR lines were assumed completely inbred. Genotypes of MS and NR were used for the calculation of additive and epistatic genomic relationship matrices for heterotic group 1. If an MS and NR lines had the same genotypes for all SNPs, it was only included once in the relationship matrices.

Thus, the M2 model was:


(6) $$\begin{array}{l} y=Xb+(T_{7}+T_{8}){ɡ}_{A_{\left(1,1\right)}}+T_{2}{ɡ}_{A_{\left(2\right)}}+T_{3}{ɡ}_{D}+(T_{7}+T_{8}){ɡ}_{AA_{\left(1,1\right)}}+T_{2}{ɡ}_{AA_{\left(2\right)}}+T_{3}{ɡ}_{AA_{\left(3\right)}}\\ +(T_{7}+T_{8})r_{\left(1,1\right)}+T_{2}r_{\left(2\right)}+T_{3}r_{\left(3\right)}+T_{4}k+T_{5}l+T_{6}m+e \end{array}$$

where ***y*** is the vector of phenotypes of the three-way hybrids; ***T*** *_7_* and ***T*** *_8_* are design matrices for MS and NR, respectively; ${ɡ}_{A_{\left(1,1\right)}}$ , ${ɡ}_{AA_{\left(1,1\right)}}$ , and $r_{A_{\left(1,1\right)}}$ are vectors of additive, epistatic, and residual genetic effects for both MS and NR, respectively, with ${ɡ}_{A_{\left(1,1\right)}}∼\;N\left(0,\frac{1}{2}G_{A_{\left(1,1\right)}}\sigma_{A_{\left(1,1\right)}}^{2}\right)$ , ${ɡ}_{AA_{\left(1,1\right)}}∼N\left(0,G_{AA_{\left(1,1\right)}}\sigma_{AA_{\left(1,1\right)}}^{2}\right)$ , and $r_{\left(1,1\right)}∼\;N\left(0,I_{r_{\left(1,1\right)}}\sigma_{r_{\left(1,1\right)}}^{2}\right)$ , where $\sigma_{A_{\left(1,1\right)}}^{2}$ , $\sigma_{AA_{\left(1,1\right)}}^{2}$ and $\sigma_{r_{\left(1,1\right)}}^{2}\;$ are additive, within-group epistatic and residual genetic variances for MS and NR, and $G_{A_{\left(1,1\right)}}$ and $G_{AA_{\left(1,1\right)}}$ are additive and epistatic genomic relationship matrices, and $I_{r_{\left(1,1\right)}}$ is an identity matrix. $G_{A_{\left(1,1\right)}}$ was scaled by ½ to account for the first cross between MS and NR, which produced the two-way cross. Additionally, ***M*** *_2_*, which was used in the calculation of the additive genomic relationship matrix for group 2 ( $G_{A_{\left(2\right)}}$ ) now included heterozygous genotypes *B_2_b_2_* coded as 0.5. The marker matrix for the dominance relationship matrix, ***W*** *_2_*, was extended to account for heterozygous genotypes in the two-way crosses and in the R lines, which now have twelve possible crossing combinations instead of four in M1 (**Table 2**). The additive-by-additive epistatic relationship matrix between lines in group 1 and 2 was calculated as:


(7) $$G_{AA_{\left(3\right)}}=\frac{(T_{7}+T_{8})G_{A_{\left(1,1\right)}}{\left(T_{7}+T_{8}\right)}^{'}⨀\;T_{2}G_{A_{\left(2\right)}}T_{2}^{'}}{tr\left((T_{7}+T_{8})G_{A_{\left(1,1\right)}}{\left(T_{7}+T_{8}\right)}^{'}⨀\;T_{2}G_{A_{\left(2\right)}}T_{2}^{'}\right)/n_{H}}$$

In M3, the same model parameters were used as for M2 (Equation 6), but now every parental line (MS, NR, two-way crosses, and R) could have arbitrary levels of inbreeding. Therefore, ***M***_1_, which was used in the calculation of the additive genomic relationship matrix for MS and NR ( $G_{A_{\left(1,1\right)}}$ ) included heterozygous genotypes *B_1_b_1_* coded as 0.5. The marker matrix for the dominance relationship matrix, ***W*** *_3_*, was further extended to account for heterozygous genotypes in all parental lines, which now have 27 possible crossing combinations (**Table 2**).

### 2.3 Estimation of variance components and heritabilities

Variance components for the random effects included in M1, M2, and M3 were estimated by restricted maximum likelihood using the software package DMU (Madsen and Jensen, 2013). Estimated genetic variances were multiplied with D_K_ (the mean of the diagonal of the respective relationship matrix minus the overall mean of the matrix) in order to account for the lack of Hardy-Weinberg equilibrium (Legarra, 2016; Vitezica et al., 2017). Narrow-sense heritabilities were calculated as the sum of additive genetic variances divided by total phenotypic variance, and broad-sense heritabilities were calculated as sum of additive and non-additive genetic variances divided by total phenotypic variance. Heritabilities were calculated both at plot level and at entry mean level of the three-way hybrids, i.e. based on the mean of all plot records for each three-way hybrid.

Phenotypic variance at plot level, ${\hat{\sigma }}_{p_{plot}}^{2}$ , was calculated as:


(8) $${\hat{\sigma }}_{p_{plot}}^{2}={\hat{\sigma }}_{{ɡ}_{A}}^{2}+{\hat{\sigma }}_{{ɡ}_{AA}}^{2}+{\hat{\sigma }}_{{ɡ}_{D}}^{2}+{\hat{\sigma }}_{r}^{2}+{\hat{\sigma }}_{k}^{2}+{\hat{\sigma }}_{l}^{2}+{\hat{\sigma }}_{m}^{2}+{\hat{\sigma }}_{e}^{2}$$

where ${\hat{\sigma }}_{{ɡ}_{A}}^{2}$ is the estimated sum of additive genetic variances for group 1 and 2, ${\hat{\sigma }}_{{ɡ}_{AA}}^{2}$ is the estimated sum of epistatic genetic variances within and across group 1 and 2, ${\hat{\sigma }}_{{ɡ}_{D}}^{2}$ is estimated dominance genetic variance, ${\hat{\sigma }}_{r}^{2}$ is the estimated sum of residual genetic variances of group 1, group 2 and of the hybrids, ${\hat{\sigma }}_{k}^{2}$ , ${\hat{\sigma }}_{l}^{2},$ and ${\hat{\sigma }}_{m}^{2}$ are estimated variances for the year x location interactions defined above for M1, and ${\hat{\sigma }}_{e}^{2}$ is estimated residual variance.

Phenotypic variance at entry mean level of the three-way hybrids, ${\hat{\sigma }}_{p_{entry}}^{2}$ , was calculated as:


(9) $${\hat{\sigma }}_{p_{entry}}^{2}={\hat{\sigma }}_{{ɡ}_{A}}^{2}+{\hat{\sigma }}_{{ɡ}_{AA}}^{2}+{\hat{\sigma }}_{{ɡ}_{D}}^{2}+{\hat{\sigma }}_{r}^{2}+\frac{{\hat{\sigma }}_{k}^{2}}{n_{k}}+\frac{{\hat{\sigma }}_{l}^{2}}{n_{l}}+\frac{{\hat{\sigma }}_{m}^{2}}{n_{m}}+\frac{{\hat{\sigma }}_{e}^{2}}{n_{e}}$$

where *n_k_* is average number of year x location observed per two-way cross, *n_l_* is average number of year x locations observed per R line, *n_m_* is average number of observations per year x location x treatment interaction (only included for rye), and *n_e_* is average number of observations per three-way hybrid.

### 2.4 Cross-validation strategies and predictive abilities

Predictive abilities of the models were evaluated using four different leave-one-out cross-validation strategies. Phenotypes of three-way hybrids were left out from the training set based on their parental components and predicted based on the remaining data. In the four cross-validations, the phenotypes were left out based on I) the maternal two-way cross, II) the paternal R line, III) the specific combination of the two parents (three-way hybrid), or IV) based on the breeding cycle of the R lines. Predictive abilities were then defined as the correlation between estimated genetic effects and the phenotype corrected for all other effects, which is equivalent to the genetic effects estimated from the full model plus the residual effects. The strategies were chosen to evaluate predictive abilities for total genetic values, for GCA of each parental line, and for SCA. For total genetic values, correlations were calculated based on the sum of all genetic effects, and for GCA and SCA, correlations were based only on the effects of the component that was left out in the cross-validation strategy. GCA was estimated as the sum of additive genetic effects and with-in group epistasis of parental components from each heterotic group, and SCA was estimated as across-group epistasis and dominance of the three-way hybrids. Furthermore, correlations were calculated at plot level and at entry mean level of the three-ways hybrids.

## 3 Results

### 3.1 Phenotyping and genotyping

**Figure 1:**
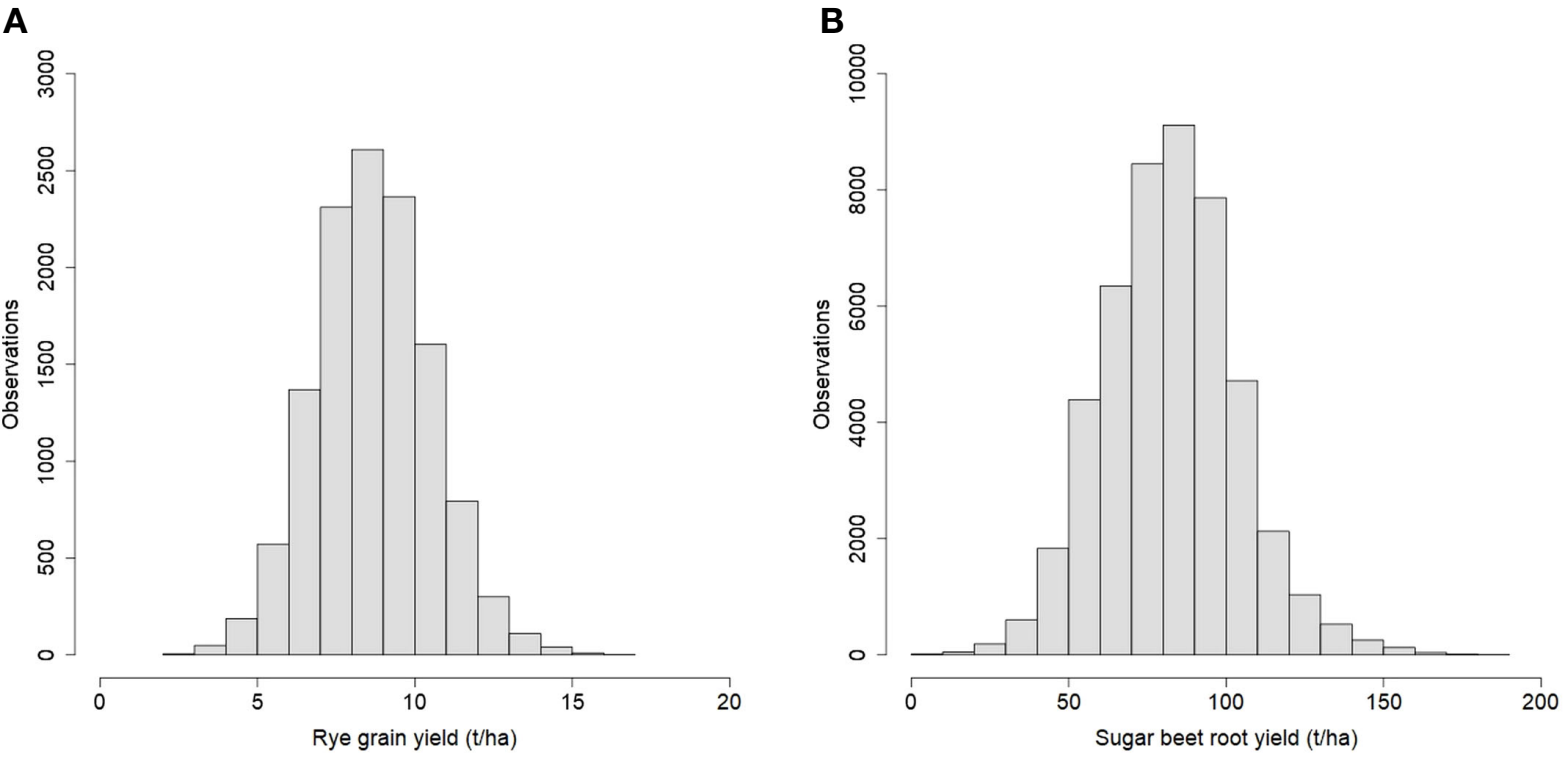
Histograms of phenotypic observations for **(A)** rye grain yield and **(B)** sugar beet root yield.

For rye, 570 three-way hybrids were phenotyped for grain yield with a total of 12,326 plot observations from seven years. For sugar beet, 657 three-way hybrids were phenotyped for root yield with a total of 47,703 plot observations from eleven years. The distributions of the phenotypes are shown in **Figure 1**, and for both traits, they were approximately normally distributed. The average rye grain yield was 8.7 t/ha with a coefficient of phenotypic variation of 21.1%, and the average sugar beet root yield was 82.4 t/ha with a coefficient of phenotypic variation of 25.8%.

The number of parental components used for producing the hybrids are shown in **Table 1**. The MS, NR and R lines had been inbred for several generations and had a high degree of homozygosity based on SNP markers (mean from 88 to 96%, **Table 3**). The homozygosity based on the SNPs might be different from the homozygosity of QTL, because each QTL is most likely not in complete linkage disequilibrium with one SNP. The two-way crosses were produced by crossing MS and NR lines belonging to the same heterotic group, and their mean homozygosity estimated from the parental genotypes was therefore relatively high (70% and 86% for sugar beet and rye, respectively). Each two-way cross was a mix of plants that were homozygous and plants that were heterozygous for SNPs that were heterozygous in at least one of the parental lines (mean of 7% and 23% of the SNPs for rye and sugar beet, respectively).

**Table 3:** Mean homozygosity and heterozygosity of parental components estimated based on SNP markers.

Crop	Component	Mean homozygosity, %	Mean heterozygosity, %
Rye	MS+NR lines	96 (0.8)	4 (0.8)
	R lines	95 (0.2)	5 (0.2)
	Two-way crosses	86 (0.8)	14 (0.8)
Sugar beet	MS+NR lines	88 (0.8)	12 (0.8)
	R lines	88 (0.5)	12 (0.5)
	Two-way crosses	70 (0.5)	30 (0.5)

For two-way crosses, estimations were based on genotypes of their parental components. Standard errors are in parentheses.

**Figure 2:**
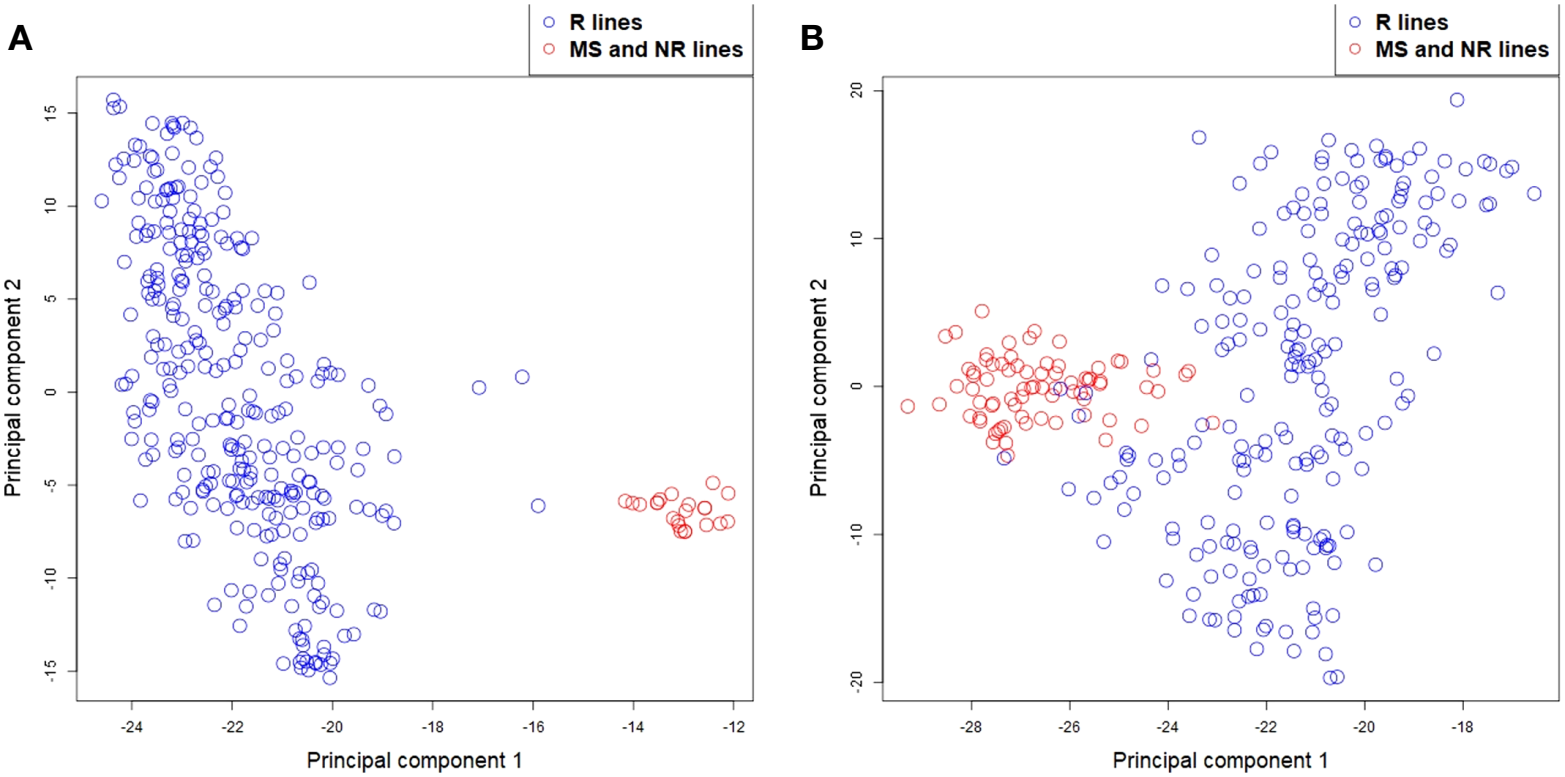
Principal component analysis for **(A)** rye and **(B)** sugar beet MS, NR (red circles) and R lines (blue circles) based on their SNP markers.

Plots of the first two principal components from a principal component analysis of the SNP genotypes of the parental lines (explaining 39.6% and 5.2% of the variance for rye, and 55.4% and 4.4% for sugar beet) showed that the MS and NR lines were located together in one small group for both crops, while the R lines formed another and more diverse group (**Figure 2**). For rye, the two groups were clearly separated, while there was a small overlap between the groups for sugar beet.

### 3.2 Genetic variances and heritabilities

**Figure 3:**
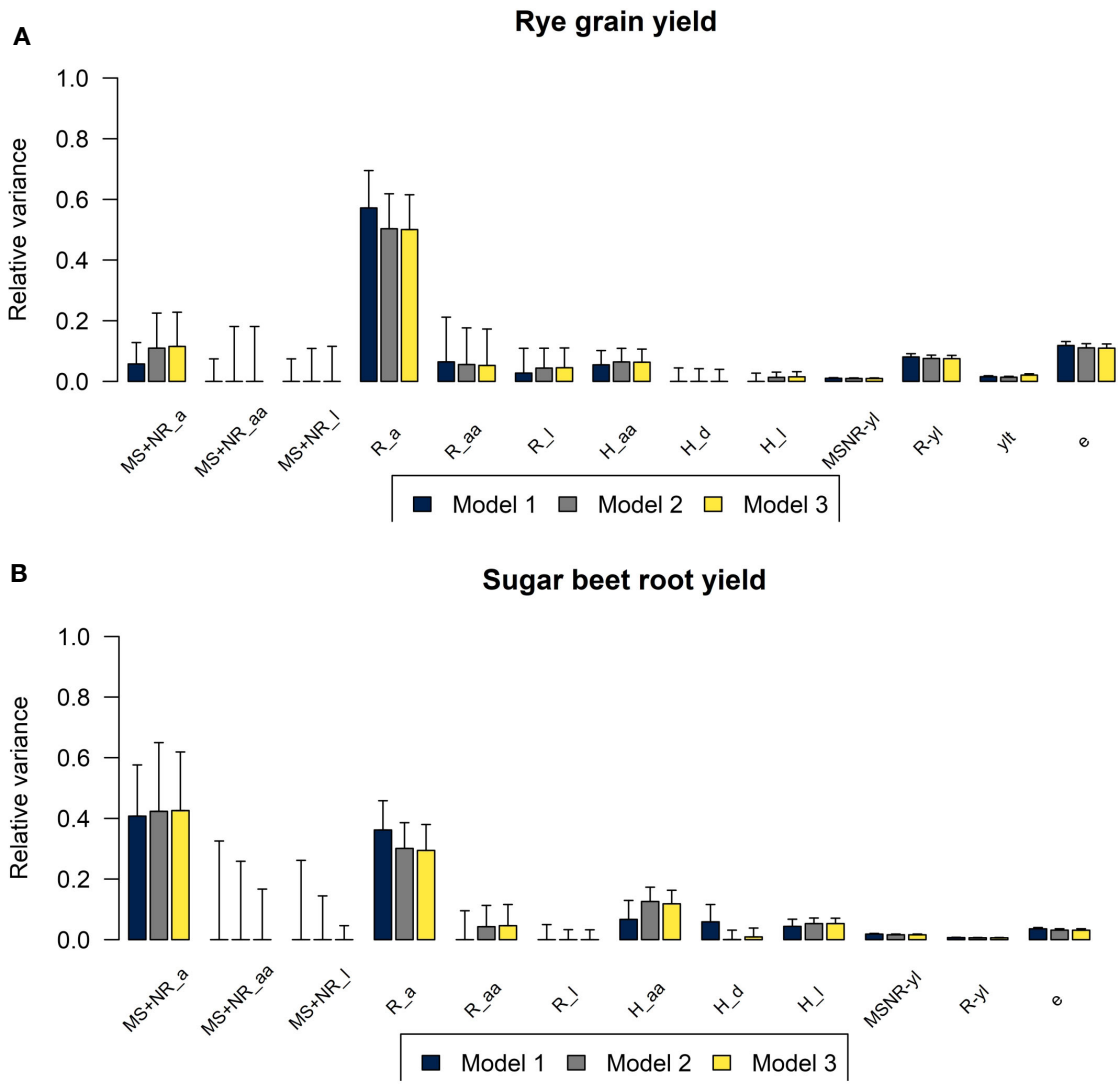
Plots of relative variance components for **(A)** rye grain yield and for **(B)** sugar beet root yield estimated using M1 (blue), M2 (grey), and M3 (yellow). Variances of additive (a), additive-by-additive (aa), dominance (d) and residual genetic effects (l) in MS+NR, R lines and in three-way hybrids (H), interaction effects between parental components and year-location (MSNR-yl and R-yl), year-location-treatment (ylt) and residuals (e) shown as proportions of total phenotypic variance at entry mean level.

The estimated variance components for the three genomic prediction models M1, M2, and M3 that differed in the assumptions about inbreeding of the parental components are shown in **Figure 3**. For both crops, the differences in the estimated variances were relatively small when comparing M1, M2, and M3. For M1, the additive genetic variance for R lines was higher, and the additive genetic variance for MS and NR was lower, compared to the estimated variances for M2 and M3. For grain yield in rye, the majority of the phenotypic variance of the three-way hybrids could be explained by additive genetic variance in the R lines (57% for M1 and 50% for M2 and M3). Additive genetic variance of MS and NR and epistatic variances of R lines and of three-way hybrids explained similar, but smaller proportions of the total variance, ranging from 5% to 11%. For sugar beet root yield, additive genetic variances explained large proportions of the total variance for both MS and NR and for R (41% and 36%, respectively, based on M1, and 43% and 30% based on M2 and M3). For M1, variances for epistatic and dominance effects in the three-way hybrids explained equal proportions, while the epistatic variance explained a higher proportion in M2 and M3, and the dominance variance in the three-way hybrids explained less than 1%.

The estimated heritabilities of both traits were high at entry mean level and intermediate at plot level (**Table 4**). The heritabilities estimated based on the three models were very similar, however the broad-sense heritabilities were slightly higher based on M2 and M3 than based on M1. For sugar beet, the differences between the broad-sense and narrow-sense heritabilities were slightly larger based on M2 and M3 than based on M1. Genetic variances of GCA of MS+NR lines were considerably lower than variances of GCA of R lines for rye, while variances of GCA of MS+NR and of R lines were similar for sugar beet. Genetic variances of SCA were lower than variances of GCA for both crops.

**Table 4:** Broad-sense (H^2^) and narrow-sense (h^2^) heritabilities estimated from M1, M2 and M3 at entry mean level of the three-way hybrids or at plot level, and estimated genetic variances of GCA for each heterotic group ( $\sigma_{GCA_{\left(MS+NR\right)}}^{2}$ and $\sigma_{GCA_{\left(R\right)}}^{2}$ ) and SCA ( $\sigma_{SCA}^{2}$ ).

	Rye, grain yield			Sugar beet, root yield		
	Model 1	Model 2	Model 3	Model 1	Model 2	Model 3
H^2^, entry mean	0.77 (0.03)	0.79 (0.03)	0.79 (0.03)	0.94 (0.01)	0.95 (0.01)	0.95 (0.01)
h^2^, entry mean	0.57 (0.13)	0.58 (0.15)	0.59 (0.14)	0.77 (0.18)	0.75 (0.22)	0.74 (0.19)
H^2^, plot	0.15 (0.02)	0.16 (0.02)	0.16 (0.02)	0.21 (0.02)	0.24 (0.03)	0.24 (0.03)
h^2^, plot	0.11 (0.03)	0.12 (0.04)	0.12 (0.03)	0.17 (0.05)	0.19 (0.07)	0.19 (0.06)
$\sigma_{GCA_{\left(MS+NR\right)}}^{2}$	1.01*10^-2^ (1.18*10^-2^)	2.03*10^-2^ (2.90*10^-2^)	2.13*10^-2^ (2.73*10^-2^)	5.87 (4.27)	7.87 (3.15)	8.10 (2.62)
$\sigma_{GCA_{\left(R\right)}}^{2}$	11.51*10^-2^ (2.45*10^-2^)	10.60*10^-2^ (2.35*10^-2^)	10.62*10^-2^ (2.36*10^-2^)	5.28 (1.29)	5.90 (1.34)	5.90 (1.33)
$\sigma_{SCA}^{2}$	0.98*10^-2^ (0.57*10^-2^)	1.51*10^-2^ (0.65*10^-2^)	1.39*10^-2^ (0.66*10^-2^)	1.94 (0.48)	1.67 (0.46)	1.72 (0.46)

Standard errors are in parentheses.

### 3.3 Genomic predictions

**Figure 4:**
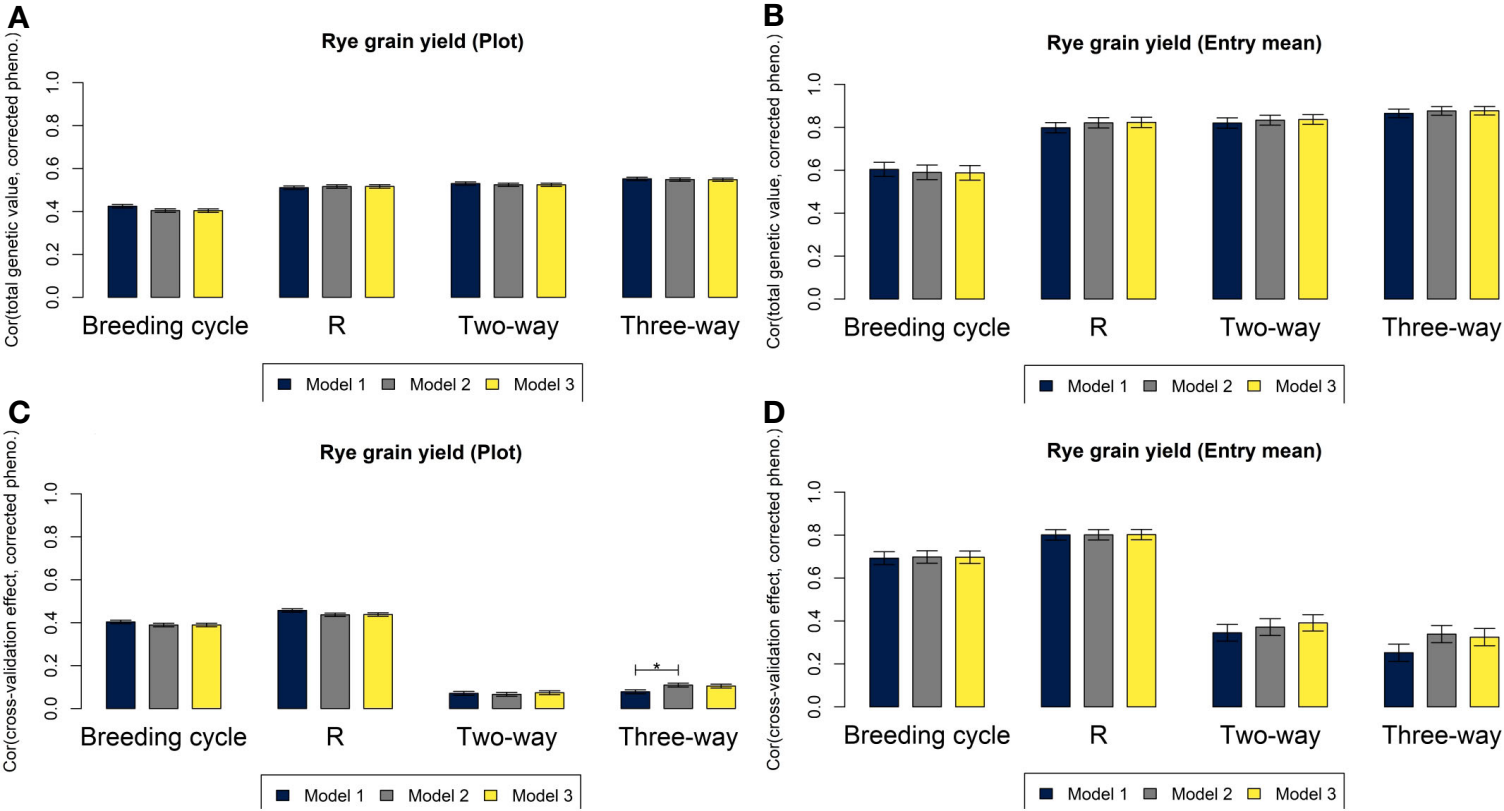
Correlations for grain yield in rye based on M1 (blue), M2 (grey) and M3 (yellow) between total genetic values and phenotypes corrected for non-genetic effects at plot level **(A)** and at entry mean level **(B)**, and between the genetic effects of the component left out in each of the four cross-validations and phenotypes corrected for other effects at plot level **(C)** and at entry mean level **(D)**. Asterisks above the bars represent significant differences between the correlations (*p*-value< 0.05/3).

**Figure 5:**
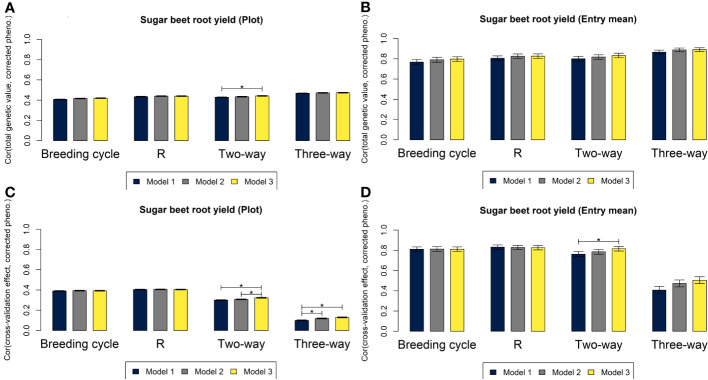
Correlations for root yield in sugar beet based on M1 (blue), M2 (grey) and M3 (yellow) between total genetic values and phenotypes corrected for non-genetic effects at plot level **(A)** and at entry mean level **(B)**, and between the genetic effects of the component left out in each of the four cross-validations and phenotypes corrected for other effects at plot level **(C)** and at entry mean level **(D)**. Asterisks above the bars represent significant differences between the correlations (*p*-value< 0.05/3).

For all three genomic prediction models, the four cross-validation strategies resulted in high predictive abilities of hybrid performance at plot level and especially at entry mean level for both rye and sugar beet (**Figures 4**, **5**). Prediction accuracies of total genetic values were up to 0.55 at plot level and 0.88 at entry mean level for M3 based on the leave three-way hybrid out cross-validations for rye and 0.47 at plot level and 0.89 at entry mean level for sugar beet. The prediction of non-additive effects in the three-way hybrids (SCA) resulted in lower predictive abilities of 0.10 at plot level and 0.32 at entry mean level for M3 for rye and of 0.13 at plot level and 0.50 at entry mean level for sugar beet. Differences between predictive abilities at plot level and at entry mean level were larger for sugar beet than for rye due to more observations per hybrid for sugar beet (average of 21.6 observations for rye and average of 72.6 observations for sugar beet). The differences between the predictions based on the three models were very small, and in most cases, M2 and M3 performed equally well or slightly better than M1. The largest significant differences between the models were for prediction of SCA, where the predictive ability increased from 0.10 at plot level for M1 to 0.13 for M3 for sugar beet and from 0.08 for M1 to 0.11 for M2 for rye. Differences in predictive abilities based on the four cross-validation strategies were mainly found for predictions of GCA or SCA rather than for prediction of total genetic values (**Figures 4**, **5C, D**). In rye, the predictive ability was lower for GCA of MS+NR lines and for SCA than for GCA of R lines. In sugar beet, the predictive ability was lower for SCA than for GCA of both parental groups. The leave-breeding cycle-out cross-validation resulted in lower predictive abilities of total genetic values, particularly for rye.

## 4 Discussion

### 4.1 Genetic variances and heritabilities

Genetic variance components of additive, epistatic and dominance deviations were estimated based on the genomic models. For rye, the majority of genetic variance for grain yield was due to additive effects from the R lines, while variances of additive genetic effects of the maternal lines and of non-additive genetic deviations were low. The reason for the low genetic variance of maternal lines might be that the tested three-way hybrids were produced from a relatively small number of different maternal lines, and there was less genetic variation between these lines based on the principal component analysis of the SNPs compared to the variation between R lines (**Figure 2**). For sugar beet, the number of maternal lines was higher, and there was more genetic variation between them based on the principal component analysis of the SNPs and also based on the estimated genetic variances for root yield. For both rye and sugar beet, genetic variance of SCA was considerably lower than variance of GCA, which is in agreement with other studies of yield in hybrid crops (Technow et al., 2014; Wang et al., 2017; Werner et al., 2018; González-Diéguez et al., 2021). However, this does not mean that the overall heterotic effects in the hybrids are small, but that the variance for these effects are low between the hybrids compared to the variance of additive genetic effects or that part of non-additive variances is captured as additive (Reif et al., 2007; Huang and Mackay, 2016).

The entry mean heritabilities for rye grain yield and sugar beet root yield were considerably higher than heritabilities at plot level due to a high average number of observations per hybrid in the datasets. The residual variances were therefore relatively low, when corrected for the average number of observations. Similarly, the correlations between estimated genetic effects and the corrected phenotypes at entry mean level were also higher than correlations based on corrected phenotypes at plot level due to the high average number of observations per hybrid. Entry mean heritabilities in the same high range as in the current study have been reported in previous studies of grain yield in rye (Wang et al., 2014; Auinger et al., 2016; Schulthess et al., 2016). For sugar beet, the differences between broad- and narrow-sense heritabilities based on the three genomic models indicated that a slightly smaller part of the genetic variance was captured as additive in models M2 and M3 accounting for incomplete inbreeding compared to M1. The degree of mean heterozygosity in the parental lines of the hybrids were higher in sugar beet than in rye (**Table 3**). Thus, larger differences between the estimated variance components and between the predictive abilities of the three models were expected for sugar beet than for rye. The estimates of residual genetic variance were slightly higher for R lines in rye and for three-way hybrids in sugar beet based on M2 and M3 than M1, which could be due to the heterozygosity of SNPs in the parental lines that was included in M2 and M3. Hybrids produced from parents that are heterozygous for some loci, will be a mixture of plants that are homozygous and plants that are heterozygous for those loci, and thereby the effects of the loci are difficult to estimate correctly.

The models M1, M2, and M3 are equivalent if all parental components are completely inbred. In M2 and M3, the genotypes of MS and NR parental lines were directly used in calculations of the genomic relationship matrices, and the heterozygosity of the two-way crosses between them were accounted for. Unlike in M1, which was developed for two-way hybrids. However, the differences between the variance components estimated from the three models were small, especially for rye, due to the low degree of heterozygosity in both parental groups of the evaluated breeding material. Larger differences between the estimates of the models would be expected for breeding programs with higher degrees of heterozygosity in the parental components, e.g. if synthetics are used as restorers for top-cross hybrids (Siekmann et al., 2021; Hackauf et al., 2022).

### 4.2 Partitioning of genetic variance

The sum of genetic variances estimated based on the three models were similar. However, for M1 the additive variance of the MS+NR lines was lower, and the additive variance of R lines was higher than for M2 and M3. In model M1 that assumes complete inbreeding of parental lines, the genomic relationship matrices all have a mean diagonal equal to 1 and an overall mean very close to 0. Therefore, the estimates of the variance components can be interpreted as genetic variances, although the genotypes are not in Hardy-Weinberg equilibrium (Legarra, 2016; González-Diéguez et al., 2021). However, when including heterozygous marker genotypes in M2 and M3, this no longer holds true. Therefore, estimated variance components were be multiplied with D_k_ (the mean of the diagonal of the respective relationship matrix minus the overall mean of the matrix) to be practically interpretable as genetic variances of the groups in the breeding programs (Legarra, 2016; Vitezica et al., 2017). Correct partitioning of genetic variances into additive and non-additive variances is important in order to make informed decisions in breeding programs that might affect short- or long-term genetic gain of GCA and SCA (Allier et al., 2019). If all QTL for additive and non-additive effects are assumed to be in linkage equilibrium, genetic variances can be estimated orthogonally, and it should thereby be possible to partition genetic variances correctly into additive, epistatic and dominance variances using the genomic models (**Appendix 1**) (Cockerham et al., 1954). However, when using empirical data, the assumption of linkage equilibrium is rarely true, and therefore estimated variances of different genetic effects might be correlated. Consequently, the partitioning of genetic variances may change depending on which parameters are included in the models (González-Diéguez et al., 2021; Raffo et al., 2022). Thus, estimated genetic variances should be carefully interpreted, as they might not completely reflect the corresponding underlying biological additive and non-additive gene actions (Huang and Mackay, 2016).

### 4.3 Dominance and epistatic deviations

High predictive abilities have been reported in studies of hybrid rye (Wang et al., 2014; Auinger et al., 2016) and sugar beet (Hofheinz et al., 2012; Würschum et al., 2013) that used genomic prediction models, where non-additive effects were not explicitly included. This indicates that there is low variance for the non-additive genetic effects or that non-additive genetic effects can be partially captured as additive (Huang and Mackay, 2016; Vitezica et al., 2017), which is in accordance with the results of the current study. Including dominance in genomic prediction models has been shown to result in similar or improved predictive abilities. The improvement depends on the ratio between additive and dominance variances of the trait and on the variability of inbreeding in the studied populations (Nishio and Satoh, 2014; Zhao et al., 2014; Duenk et al., 2017; Wang et al., 2017; Werner et al., 2018; Ramstein et al., 2020; Roth et al., 2022).

Different conclusions have been reached in studies of the effect of including epistasis in genomic prediction models. For wheat, improvements in predictive abilities have been reported when epistasis was included in addition to additive genetic effects (Jiang and Reif, 2015; He et al., 2016; Raffo et al., 2022). For maize, predictive abilities were reported to be similar for models with or without inclusion of epistasis (Jiang and Reif, 2015; González-Diéguez et al., 2021). For other species, examples of reductions in predictive abilities have been reported, when epistasis was modelled (Lorenzana and Bernardo, 2009; Forneris et al., 2017). The parameterization of genetic effects and the degree of linkage disequilibrium between markers and QTL can affect how genetic effects are captured and partitioned in the models (Huang and Mackay, 2016; Schrauf et al., 2020). Thus, including epistasis can potentially improve prediction models, but the effect depends on the species, the genomic relationships of the studied populations, the genetic architecture of the trait, and on marker densities.

Dominance and epistatic deviations were included in the genomic prediction models of the current study. Accurate partitioning of the genetic variances of these non-additive deviations is challenging, because their genomic relationship matrices were highly correlated both in the current study and in González-Diéguez et al. (2021). However, the non-additive genetic variances were small compared to the additive genetic variances. Besides potentially improving predictive abilities, an advantage of including non-additive deviations in the models is that it enables predictions of the best combinations of parental components to produce hybrids. This can especially be helpful for hybrid breeding programs, where there is large genetic variance for SCA.

### 4.4 Cross-validation strategies and predictive abilities

Four different cross-validation strategies were used to evaluate the predictive ability of the three models for GCA, SCA and total genetic value in different scenarios. Prediction of GCA of parental components is important for selection of lines within heterotic groups, and prediction of SCA and total genetic value of hybrids is important for selection of the optimal combinations of parental lines. The cross-validation strategies leave two-way cross out and leave R line out were used to study the predictive abilities for GCA of untested parental components from each heterotic group. The leave three-way out strategy was used to study predictive ability for SCA, when parental components had been tested in other combinations. These cross-validations reflect the potential of the models in scenarios, where half-sibs and full-sibs of untested components are included in the training set. However, breeders are often interested in predicting genetic values of untested lines based only on previous breeding cycles (Auinger et al., 2016). Thus, leave breeding cycle out cross-validations were used to study the predictive abilities for total genetic values of hybrids and for GCA of R lines within each breeding cycle based on the remaining cycles.

Predictive abilities based on the leave breeding cycle out cross-validations were lower than predictive abilities based on the other cross-validation strategies, because larger parts of the phenotypic data were left out of the training set, and because the genomic relationships between components in training and test sets were lower. However, the reduction in predictive abilities was not as large as in other studies, where similar cross-validation strategies were used on data from barley and wheat breeding programs (Nielsen et al., 2016; Kristensen et al., 2018; Raffo et al., 2022). A reason for this could be that breeding programs for hybrid crops mainly use internally developed lines as crossing parents for new breeding cycles, while breeding programs for line cultivars commonly include cultivars developed in external breeding programs as crossing parents in addition to their internally developed lines (Lüttringhaus et al., 2020). Thus, genomic relationships between lines in training set and lines from new, untested breeding cycles would be expected to be higher within hybrid breeding programs. Another reason could be the high number of breeding cycles in the training data. The reduction in predictive ability based on the leave breeding cycle out cross-validation was lower for sugar beet than for rye, which could indicate that having data from a higher number of breeding cycles would lead to higher predictive abilities (Auinger et al., 2016; Bernal-Vasquez et al., 2017). However, when many breeding cycles are included, predictive abilities based on this cross-validation strategy might be inflated if some lines have been used as crossing parents for new breeding cycles and data based on their offspring was included in the training set. Furthermore, predicted genetic values might be inflated, because linkage disequilibrium between markers and QTL erodes over several generations (Boichard et al., 2022).

### 4.5 Models accounting for incomplete inbreeding

The predictive abilities for total genetic values of hybrids were equally high for the three genomic models for both rye and sugar beet. The largest differences in predictive abilities between the models were for GCA of MS+NR lines and for SCA, while predictive abilities for GCA of R lines were very similar for the models. This was as expected since R lines were almost completely homozygous and thus better comply with the assumption of complete inbreeding in M1. The two-way crosses were more heterozygous, and the extended models M2 and M3 could therefore capture GCA of MS+NR and SCA more accurately than M1. Even though the differences between predictive abilities of the three models were quite small, it would be advantageous to use M2 or M3 over M1 for breeding of three-way hybrids, because these models enable prediction of GCA for both MS and NR lines and not only for their two-way crosses as in M1. Additionally, models that account for incomplete inbreeding can be useful in a wider range of real or simulated breeding schemes, where lines are more heterozygous than in the datasets used here. For example, genetic values might be estimated more accurately for lines in early generations, before they have reached a high degree of homozygosity (Bernal-Vasquez et al., 2017). Thereby, lines can be selected as crossing parents earlier, and the generation time of breeding cycles can be reduced, which could lead to higher genetic gains.

The three-way rye hybrids evaluated in the current study were based on the Gülzow (G) type cytoplasmic male sterility (CMS) (Melz et al., 2003; Vendelbo et al., 2021b). The frequency of non-restoration alleles for the G type system is low in Central European rye germplasm, which makes it challenging to increase genetic variation of NR lines for breeding (Łapiński and Stojałowski, 2003; Hackauf et al., 2022). For the Owen type CMS in sugar beet, non-restoration alleles are rare in most populations as well (Moritani et al., 2013). Consequently, the genetic variation of the heterotic group of MS and NR lines was low compared to the heterotic group of R lines in both crops, and the degree of homozygosity of the maternal two-way hybrids were high, especially in rye. Thereby, the experimental three-way hybrids resemble two-way hybrids to a large extent. The predominant hybridization system in rye is based on the Pampa (P) type CMS. In contrast to the G type CMS system, non-restoration alleles are common, while restoration alleles are rare for the P type system. Additionally, synthetic restorers from two inbred lines are used for the production of the commercial top-cross hybrids. Thus, the average degree of heterozygosity in restorers of the P type based breeding systems is higher than in the R lines of the current study (Siekmann et al., 2021). The models accounting for incomplete inbreeding could thereby be advantageous to use, not only for three-way hybrids programs, but also for breeding programs based on the P type CMS system.

## 5 Conclusion

Three genomic models for predicting hybrid performance were evaluated based on data from two commercial breeding programs. The models can be used to predict GCA of parental lines within each of two heterotic groups and to predict SCA of realized and potential three-way hybrids. Estimated genetic variances of GCA (additive and within-group epistasis) were considerably larger than variances of SCA (across-group epistasis and dominance) for both grain yield in rye and root yield in sugar beet. For rye, variance of GCA of R lines were larger than variance of GCA of MS+NR lines, while variances of GCA of both parental groups were more similar for sugar beet. Average levels of heterozygosity in parental components were low, and therefore, the differences between the three models were small. The predictive ability of model M1, which assumes complete inbreeding in parental lines, was similar to or lower than the predictive ability of the extended models M2 and M3 accounting for incomplete inbreeding in parental lines of three-way hybrids. The predictive abilities of the three models were similar for predicting total genetic values of hybrids and for GCA of R lines. For prediction of GCA of MS+NR lines in sugar beet and of SCA in both crops, predictive abilities significantly improved when using the extended models compared to using M1. Promising NR lines can potentially be selected for producing new MS lines via backcrossing at earlier stages in the breeding programs, because the extended models enable prediction of GCA for both MS and NR lines. Additionally, NR or R lines with high GCA can be selected as crossing parents for new breeding cycles within each heterotic group.

The predictive ability of the models was high for prediction of hybrid performance (total genetic value), and the models can therefore be a valuable tool for selecting the most promising parental lines to produce new hybrids. Due to the relatively narrow genetic variation within the heterotic group of MS+NR lines, the differences in performance of hybrids will mainly be affected by the GCA of their parental R lines for grain yield in rye. However, other traits such as flowering time, plant height, and disease resistances should also be considered when selecting the optimal combinations of parents. For root yield in sugar beet, performance of hybrids will be more equally affected by GCA of parents from both heterotic groups and to a smaller extent by the SCA of the combinations.

The models developed here are suitable for a wide range of hybrid breeding programs, where the parental lines can have any level of inbreeding. Thus, the genomic prediction models might improve breeding programs for hybrid crops by facilitating selection of lines within heterotic groups as well as selection of best combinations of lines across groups for the production of new hybrids.

## Data availability statement

The original contributions presented in the study are included in the article/**Supplementary Material**. Further inquiries can be directed to the corresponding author.

## Author contributions

JJ, AJ, JH, LR, PSn, JO, PSa, and PK contributed to conception and design of the study. Data was collected and curated by PSa, JO, LR, PSn, DF, PK, and MM. PK and PSa performed statistical analysis. PK, PSa, JJ, DF, and TC contributed to choosing statistical methods and interpreting results. PK wrote the first draft of the manuscript, and all authors contributed to manuscript revision.
