# Supplementary material for: Prediction of additive, epistatic, and dominance effects using models accounting for incomplete inbreeding in parental lines of hybrid rye and sugar beet
Source: Front Plant Sci. 2023 Nov 2;14:1193433. doi: 10.3389/fpls.2023.1193433 (PMC10756082; doi:10.3389/fpls.2023.1193433)
Supplement: Supplementary file 5 [file DataSheet_5.docx]

# Appendix 1

## Dominance deviations in three-way hybrids

Table A1. Frequencies of genotypes in a three-way hybrid population produced from parental lines of heterotic group 1 and group 2.

| **Parental genotypes*** | **Frequency** |
| --- | --- |
| *(B_1_B_1_ x B_1_B_1_) x B_2_B_2_* | ($p_{1}p_{1}+p_{1}q_{1}F_{1}$)( $p_{1}p_{1}+p_{1}q_{1}F_{1}$)( $p_{2}p_{2}+p_{2}q_{2}F_{2}$) |
| *(B_1_B_1_ x B_1_B_1_) x B_2_b_2_* | ($p_{1}p_{1}+p_{1}q_{1}F_{1}$)( $p_{1}p_{1}+p_{1}q_{1}F_{1}$)($2p_{2}q_{2}-2p_{2}q_{2}F_{2}$) |
| *(B_1_B_1_ x B_1_B_1_) x b_2_b_2_* | ($p_{1}p_{1}+p_{1}q_{1}F_{1}$)( $p_{1}p_{1}+p_{1}q_{1}F_{1}$)( $q_{2}q_{2}+p_{2}q_{2}F_{2}$) |
| *(B_1_B_1_ x B_1_b_1_) x B_2_B_2_*  *(B_1_b_1_ x B_1_B_1_) x B_2_B_2_* | ($p_{1}p_{1}+p_{1}q_{1}F_{1}$)${(2p}_{1}q_{1}-2p_{1}q_{1}F_{1})$( $p_{2}p_{2}+p_{2}q_{2}F_{2}$) |
| *(B_1_B_1_ x B_1_b_1_) x B_2_b_2_*  *(B_1_b_1_ x B_1_B_1_) x B_2_b_2_* | ($p_{1}p_{1}+p_{1}q_{1}F_{1}$)${(2p}_{1}q_{1}-2p_{1}q_{1}F_{1})$($2p_{2}q_{2}-2p_{2}q_{2}F_{2}$) |
| *(B_1_B_1_ x B_1_b_1_) x b_2_b_2_*  *(B_1_b_1_ x B_1_B_1_) x b_2_b_2_* | ($p_{1}p_{1}+p_{1}q_{1}F_{1}$)${(2p}_{1}q_{1}-2p_{1}q_{1}F_{1})$( $q_{2}q_{2}+p_{2}q_{2}F_{2}$) |
| *(B_1_B_1_ x b_1_b_1_) x B_2_B_2_*  *(b_1_b_1_ x B_1_B_1_) x B_2_B_2_* | ($p_{1}p_{1}+p_{1}q_{1}F_{1}$)${(q}_{1}q_{1}+p_{1}q_{1}F_{1})$( $p_{2}p_{2}+p_{2}q_{2}F_{2}$) |
| *(B_1_B_1_ x b_1_b_1_) x B_2_b_2_*  *(b_1_b_1_ x B_1_B_1_) x B_2_b_2_* | ($p_{1}p_{1}+p_{1}q_{1}F_{1}$)${(q}_{1}q_{1}+p_{1}q_{1}F_{1})$($2p_{2}q_{2}-2p_{2}q_{2}F_{2}$) |
| *(B_1_B_1_ x b_1_b_1_) x b_2_b_2_*  *(b_1_b_1_ x B_1_B_1_) x b_2_b_2_* | ($p_{1}p_{1}+p_{1}q_{1}F_{1}$)${(q}_{1}q_{1}+p_{1}q_{1}F_{1})$( $q_{2}q_{2}+p_{2}q_{2}F_{2}$) |
| *(B_1_b_1_ x B_1_b_1_) x B_2_B_2_* | ${(2p}_{1}q_{1}-2p_{1}q_{1}F_{1}){(2p}_{1}q_{1}-2p_{1}q_{1}F_{1})$( $p_{2}p_{2}+p_{2}q_{2}F_{2}$) |
| *(B_1_b_1_ x B_1_b_1_) x B_2_b_2_* | ${(2p}_{1}q_{1}-2p_{1}q_{1}F_{1}){(2p}_{1}q_{1}-2p_{1}q_{1}F_{1})$($2p_{2}q_{2}-2p_{2}q_{2}F_{2}$) |
| *(B_1_b_1_ x B_1_b_1_) x b_2_b_2_* | ${(2p}_{1}q_{1}-2p_{1}q_{1}F_{1}){(2p}_{1}q_{1}-2p_{1}q_{1}F_{1})$( $q_{2}q_{2}+p_{2}q_{2}F_{2}$) |
| *(b_1_b_1_ x B_1_b_1_) x B_2_B_2_*  *(B_1_b_1_ x b_1_b_1_) x B_2_B_2_* | ${(q}_{1}q_{1}+p_{1}q_{1}F_{1}){(2p}_{1}q_{1}-2p_{1}q_{1}F_{1})$( $p_{2}p_{2}+p_{2}q_{2}F_{2}$) |
| *(b_1_b_1_ x B_1_b_1_) x B_2_b_2_*  *(B_1_b_1_ x b_1_b_1_) x B_2_b_2_* | ${(q}_{1}q_{1}+p_{1}q_{1}F_{1}){(2p}_{1}q_{1}-2p_{1}q_{1}F_{1})$($2p_{2}q_{2}-2p_{2}q_{2}F_{2}$) |
| *(b_1_b_1_ x B_1_b_1_) x b_2_b_2_*  *(B_1_b_1_ x b_1_b_1_) x b_2_b_2_* | ${(q}_{1}q_{1}+p_{1}q_{1}F_{1}){(2p}_{1}q_{1}-2p_{1}q_{1}F_{1})$( $q_{2}q_{2}+p_{2}q_{2}F_{2}$) |
| *(b_1_b_1_ x b_1_b_1_) x B_2_B_2_* | ${(q}_{1}q_{1}+p_{1}q_{1}F_{1}){(q}_{1}q_{1}+p_{1}q_{1}F_{1})$( $p_{2}p_{2}+p_{2}q_{2}F_{2}$) |
| *(b_1_b_1_ x b_1_b_1_) x B_2_b_2_* | ${(q}_{1}q_{1}+p_{1}q_{1}F_{1}){(q}_{1}q_{1}+p_{1}q_{1}F_{1})$($2p_{2}q_{2}-2p_{2}q_{2}F_{2}$) |
| *(b_1_b_1_ x b_1_b_1_) x b_2_b_2_* | ${(q}_{1}q_{1}+p_{1}q_{1}F_{1}){(q}_{1}q_{1}+p_{1}q_{1}F_{1})$( $q_{2}q_{2}+p_{2}q_{2}F_{2}$) |

* Alleles *B_1_* and *b_1_* of a locus in group 1 have frequencies *p_1_* and *q_1_* = 1 – *p_1_*, respectively. Alleles *B_2_* and *b_2_* of the locus in group 2 have frequencies *p_2_* and *q_2_* = 1 – *p_2_*, respectively. F_1_ and F_2_ are the inbreeding levels of lines in group 1 and group 2, respectively.

It is assumed that MS lines are derived from NR lines via several generations of backcrossing, so that they effectively belong to the same heterotic group (group 1), and thus have same allele frequencies. In this way genetic evaluation of both MS and NR lines can benefit from genomic relationships to other MS or NR lines. A first cross between MS and NR lines is assumed. This increases the level of heterozygosity in the two-way crosses, even if the parents are fully inbred, due to the crossing of opposite homozygotes. Allele frequencies in the two-way crosses are the same as allele frequencies in the parents assuming random crossing between MS and NR lines.

Table A2. Functional genotypic values of three-way hybrids, where *a_1_* and *a_2_* are functional additive effects for *B_1_* and *B_2_*, respectively, and *d* is dominance effect of *B_1_b_2_* and *b_1_B_2_*.

| **Parental genotypes** | **Functional genotypic value** |
| --- | --- |
| *(B_1_B_1_ x B_1_B_1_) x B_2_B_2_* | $a_{2}+a_{1}$ |
| *(B_1_B_1_ x B_1_B_1_) x B_2_b_2_* | $\frac{d+a_{2}+2a_{1}}{2}$ |
| *(B_1_B_1_ x B_1_B_1_) x b_2_b_2_* | $d+a_{1}$ |
| *(B_1_B_1_ x B_1_b_1_) x B_2_B_2_*  *(B_1_b_1_ x B_1_B_1_) x B_2_B_2_* | $\frac{d+4a_{2}+3a_{1}}{4}$ |
| *(B_1_B_1_ x B_1_b_1_) x B_2_b_2_*  *(B_1_b_1_ x B_1_B_1_) x B_2_b_2_* | $\frac{2d+2a_{2}+3a_{1}}{4}$ |
| *(B_1_B_1_ x B_1_b_1_) x b_2_b_2_*  *(B_1_b_1_ x B_1_B_1_) x b_2_b_2_* | $\frac{3d+3a_{1}}{4}$ |
| *(B_1_B_1_ x b_1_b_1_) x B_2_B_2_*  *(b_1_b_1_ x B_1_B_1_) x B_2_B_2_* | $\frac{d+2a_{2}+a_{1}}{2}$ |
| *(B_1_B_1_ x b_1_b_1_) x B_2_b_2_*  *(b_1_b_1_ x B_1_B_1_) x B_2_b_2_* | $\frac{d+a_{2}+a_{1}}{2}$ |
| *(B_1_B_1_ x b_1_b_1_) x b_2_b_2_*  *(b_1_b_1_ x B_1_B_1_) x b_2_b_2_* | $\frac{d+a_{1}}{2}$ |
| *(B_1_b_1_ x B_1_b_1_) x B_2_B_2_* | $\frac{d+2a_{2}+a_{1}}{2}$ |
| *(B_1_b_1_ x B_1_b_1_) x B_2_b_2_* | $\frac{d+a_{2}+a_{1}}{2}$ |
| *(B_1_b_1_ x B_1_b_1_) x b_2_b_2_* | $\frac{d+a_{1}}{2}$ |
| *(b_1_b_1_ x B_1_b_1_) x B_2_B_2_*  *(B_1_b_1_ x b_1_b_1_) x B_2_B_2_* | $\frac{3d+4a_{2}+a_{1}}{4}$ |
| *(b_1_b_1_ x B_1_b_1_) x B_2_b_2_*  *(B_1_b_1_ x b_1_b_1_) x B_2_b_2_* | $\frac{2d+2a_{2}+a_{1}}{4}$ |
| *(b_1_b_1_ x B_1_b_1_) x b_2_b_2_*  *(B_1_b_1_ x b_1_b_1_) x b_2_b_2_* | $\frac{d+a_{1}}{4}$ |
| *(b_1_b_1_ x b_1_b_1_) x B_2_B_2_* | $d+a_{2}$ |
| *(b_1_b_1_ x b_1_b_1_) x B_2_b_2_* | $0.5(d+a_{2})$ |
| *(b_1_b_1_ x b_1_b_1_) x b_2_b_2_* | 0 |

Table A3. Deviations from the mean of the hybrid population of total genetic values of three-way hybrids (G).

| **Parental genotypes** | **Deviations from population mean E(G):**  **G** |
| --- | --- |
| *(B_1_B_1_ x B_1_B_1_) x B_2_B_2_* | $(2dp_{1}-d-a_{2})p_{2}+(-d-a_{1})p_{1}+a_{2}+a_{1}$ |
| *(B_1_B_1_ x B_1_B_1_) x B_2_b_2_* | $\frac{(4dp_{1}-2d-2a_{2})p_{2}+(-2d-2a_{1})p_{1}+d+a_{2}+2a_{1}}{2}$ |
| *(B_1_B_1_ x B_1_B_1_) x b_2_b_2_* | $(2dp_{1}-d-a_{2})p_{2}+(-d-a_{1})p_{1}+d+a_{1}$ |
| *(B_1_B_1_ x B_1_b_1_) x B_2_B_2_*  *(B_1_b_1_ x B_1_B_1_) x B_2_B_2_* | $\frac{(8dp_{1}-4d-4a_{2})p_{2}+(-4d-4a_{1})p_{1}+d+4a_{2}+3a_{1}}{4}$ |
| *(B_1_B_1_ x B_1_b_1_) x B_2_b_2_*  *(B_1_b_1_ x B_1_B_1_) x B_2_b_2_* | $\frac{(8dp_{1}-4d-4a_{2})p_{2}+(-4d-4a_{1})p_{1}+2d+2a_{2}+3a_{1}}{4}$ |
| *(B_1_B_1_ x B_1_b_1_) x b_2_b_2_*  *(B_1_b_1_ x B_1_B_1_) x b_2_b_2_* | $\frac{(8dp_{1}-4d-4a_{2})p_{2}+(-4d-4a_{1})p_{1}+3d+3a_{1}}{4}$ |
| *(B_1_B_1_ x b_1_b_1_) x B_2_B_2_*  *(b_1_b_1_ x B_1_B_1_) x B_2_B_2_* | $\frac{(4dp_{1}-2d-2a_{2})p_{2}+(-2d-2a_{1})p_{1}+d+2a_{2}+a_{1}}{2}$ |
| *(B_1_B_1_ x b_1_b_1_) x B_2_b_2_*  *(b_1_b_1_ x B_1_B_1_) x B_2_b_2_* | $\frac{(4dp_{1}-2d-2a_{2})p_{2}+(-2d-2a_{1})p_{1}+d+a_{2}+a_{1}}{2}$ |
| *(B_1_B_1_ x b_1_b_1_) x b_2_b_2_*  *(b_1_b_1_ x B_1_B_1_) x b_2_b_2_* | $\frac{(4dp_{1}-2d-2a_{2})p_{2}+(-2d-2a_{1})p_{1}+d+a_{1}}{2}$ |
| *(B_1_b_1_ x B_1_b_1_) x B_2_B_2_* | $\frac{(4dp_{1}-2d-2a_{2})p_{2}+(-2d-2a_{1})p_{1}+d+2a_{2}+a_{1}}{2}$ |
| *(B_1_b_1_ x B_1_b_1_) x B_2_b_2_* | $\frac{(4dp_{1}-2d-2a_{2})p_{2}+(-2d-2a_{1})p_{1}+d+a_{2}+a_{1}}{2}$ |
| *(B_1_b_1_ x B_1_b_1_) x b_2_b_2_* | $\frac{(4dp_{1}-2d-2a_{2})p_{2}+(-2d-2a_{1})p_{1}+d+a_{1}}{2}$ |
| *(b_1_b_1_ x B_1_b_1_) x B_2_B_2_*  *(B_1_b_1_ x b_1_b_1_) x B_2_B_2_* | $\frac{(8dp_{1}-4d-4a_{2})p_{2}+(-4d-4a_{1})p_{1}+3d+4a_{2}+a_{1}}{4}$ |
| *(b_1_b_1_ x B_1_b_1_) x B_2_b_2_*  *(B_1_b_1_ x b_1_b_1_) x B_2_b_2_* | $\frac{(8dp_{1}-4d-4a_{2})p_{2}+(-4d-4a_{1})p_{1}+2d+2a_{2}+a_{1}}{4}$ |
| *(b_1_b_1_ x B_1_b_1_) x b_2_b_2_*  *(B_1_b_1_ x b_1_b_1_) x b_2_b_2_* | $\frac{(8dp_{1}-4d-4a_{2})p_{2}+(-4d-4a_{1})p_{1}+d+a_{1}}{4}$ |
| *(b_1_b_1_ x b_1_b_1_) x B_2_B_2_* | $(2dp_{1}-d-a_{2})p_{2}+(-d-a_{1})p_{1}+d+a_{2}$ |
| *(b_1_b_1_ x b_1_b_1_) x B_2_b_2_* | $\frac{(4dp_{1}-2d-2a_{2})p_{2}+(-2d-2a_{1})p_{1}+d+a_{2}}{2}$ |
| *(b_1_b_1_ x b_1_b_1_) x b_2_b_2_* | $(2dp_{1}-d-a_{2})p_{2}+(-d-a_{1})p_{1}$ |

Table A4. Deviations from the mean of the hybrid population of statistical additive genetic effects from parental lines in heterotic group 1 and 2 (*g_A(1,1)_* and *g_A(2)_*).

| **Parental genotypes** | **Deviations from population mean E(G):**  ***g_A(1,1)_*** + ***g_A(2)_*** |
| --- | --- |
| *(B_1_B_1_ x B_1_B_1_) x B_2_B_2_* | $(1-p_{2})\alpha_{2}+(1-p_{1})\alpha_{1}$ |
| *(B_1_B_1_ x B_1_B_1_) x B_2_b_2_* | $-\frac{(2p_{2}-1)\alpha_{2}+(2p_{1}-2)\alpha_{1}}{2}$ |
| *(B_1_B_1_ x B_1_B_1_) x b_2_b_2_* | $(1-p_{1})\alpha_{1}-p_{2}\alpha_{2}$ |
| *(B_1_B_1_ x B_1_b_1_) x B_2_B_2_*  *(B_1_b_1_ x B_1_B_1_) x B_2_B_2_* | $-\frac{(4p_{2}-4)\alpha_{2}+(4p_{1}-3)\alpha_{1}}{4}$ |
| *(B_1_B_1_ x B_1_b_1_) x B_2_b_2_*  *(B_1_b_1_ x B_1_B_1_) x B_2_b_2_* | $-\frac{(4p_{2}-2)\alpha_{2}+(4p_{1}-3)\alpha_{1}}{4}$ |
| *(B_1_B_1_ x B_1_b_1_) x b_2_b_2_*  *(B_1_b_1_ x B_1_B_1_) x b_2_b_2_* | $-\frac{4p_{2}\alpha_{2}+(4p_{1}-3)\alpha_{1}}{4}$ |
| *(B_1_B_1_ x b_1_b_1_) x B_2_B_2_*  *(b_1_b_1_ x B_1_B_1_) x B_2_B_2_* | $-\frac{(2p_{2}-2)\alpha_{2}+(2p_{1}-1)\alpha_{1}}{2}$ |
| *(B_1_B_1_ x b_1_b_1_) x B_2_b_2_*  *(b_1_b_1_ x B_1_B_1_) x B_2_b_2_* | $-\frac{(2p_{2}-1)\alpha_{2}+(2p_{1}-1)\alpha_{1}}{2}$ |
| *(B_1_B_1_ x b_1_b_1_) x b_2_b_2_*  *(b_1_b_1_ x B_1_B_1_) x b_2_b_2_* | $-\frac{2p_{2}\alpha_{2}+(2p_{1}-1)\alpha_{1}}{2}$ |
| *(B_1_b_1_ x B_1_b_1_) x B_2_B_2_* | $-\frac{(2p_{2}-2)\alpha_{2}+(2p_{1}-1)\alpha_{1}}{2}$ |
| *(B_1_b_1_ x B_1_b_1_) x B_2_b_2_* | $-\frac{(2p_{2}-1)\alpha_{2}+(2p_{1}-1)\alpha_{1}}{2}$ |
| *(B_1_b_1_ x B_1_b_1_) x b_2_b_2_* | $-\frac{2p_{2}\alpha_{2}+(2p_{1}-1)\alpha_{1}}{2}$ |
| *(b_1_b_1_ x B_1_b_1_) x B_2_B_2_*  *(B_1_b_1_ x b_1_b_1_) x B_2_B_2_* | $-\frac{(4p_{2}-4)\alpha_{2}+(4p_{1}-1)\alpha_{1}}{4}$ |
| *(b_1_b_1_ x B_1_b_1_) x B_2_b_2_*  *(B_1_b_1_ x b_1_b_1_) x B_2_b_2_* | $-\frac{(4p_{2}-2)\alpha_{2}+(4p_{1}-1)\alpha_{1}}{4}$ |
| *(b_1_b_1_ x B_1_b_1_) x b_2_b_2_*  *(B_1_b_1_ x b_1_b_1_) x b_2_b_2_* | $-\frac{4p_{2}\alpha_{2}+(4p_{1}-1)\alpha_{1}}{4}$ |
| *(b_1_b_1_ x b_1_b_1_) x B_2_B_2_* | $(1-p_{2})\alpha_{2}-p_{1}\alpha_{1}$ |
| *(b_1_b_1_ x b_1_b_1_) x B_2_b_2_* | $-\frac{(2p_{2}-1)\alpha_{2}+2p_{1}\alpha_{1}}{2}$ |
| *(b_1_b_1_ x b_1_b_1_) x b_2_b_2_* | $-p_{2}\alpha_{2}-p_{1}\alpha_{1}$ |

Table A5. Deviations from the mean of the hybrid population of genetic dominance effects due to within locus interactions between alleles from heterotic group 1 and 2 (*g_D_*). Subtracting deviations in Table A4 from deviations in Table A3 gives the dominance deviations.

| **Parental genotypes** | **Deviations from population mean E(G):**  ***g_D_*** |
| --- | --- |
| *(B_1_B_1_ x B_1_B_1_) x B_2_B_2_* | $-2d\left( p_{1}-1 \right)\left( p_{2}-1 \right)$ |
| *(B_1_B_1_ x B_1_B_1_) x B_2_b_2_* | $-d\left( p_{1}-1 \right)\left( 2p_{2}-1 \right)$ |
| *(B_1_B_1_ x B_1_B_1_) x b_2_b_2_* | $-2d\left( p_{1}-1 \right)p_{2}$ |
| *(B_1_B_1_ x B_1_b_1_) x B_2_B_2_*  *(B_1_b_1_ x B_1_B_1_) x B_2_B_2_* | $-\frac{d\left( 4p_{1}-3 \right)\left( p_{2}-1 \right)}{2}$ |
| *(B_1_B_1_ x B_1_b_1_) x B_2_b_2_*  *(B_1_b_1_ x B_1_B_1_) x B_2_b_2_* | $-\frac{d\left( 4p_{1}-3 \right)\left( 2p_{2}-1 \right)}{4}$ |
| *(B_1_B_1_ x B_1_b_1_) x b_2_b_2_*  *(B_1_b_1_ x B_1_B_1_) x b_2_b_2_* | $-\frac{d\left( 4p_{1}-3 \right)p_{2}}{2}$ |
| *(B_1_B_1_ x b_1_b_1_) x B_2_B_2_*  *(b_1_b_1_ x B_1_B_1_) x B_2_B_2_* | $-d\left( 2p_{1}-1 \right)\left( p_{2}-1 \right)$ |
| *(B_1_B_1_ x b_1_b_1_) x B_2_b_2_*  *(b_1_b_1_ x B_1_B_1_) x B_2_b_2_* | $-\frac{d\left( 2p_{1}-1 \right)\left( 2p_{2}-1 \right)}{2}$ |
| *(B_1_B_1_ x b_1_b_1_) x b_2_b_2_*  *(b_1_b_1_ x B_1_B_1_) x b_2_b_2_* | $-d\left( 2p_{1}-1 \right)p_{2}$ |
| *(B_1_b_1_ x B_1_b_1_) x B_2_B_2_* | $-d\left( 2p_{1}-1 \right)\left( p_{2}-1 \right)$ |
| *(B_1_b_1_ x B_1_b_1_) x B_2_b_2_* | $-\frac{d\left( 2p_{1}-1 \right)\left( 2p_{2}-1 \right)}{2}$ |
| *(B_1_b_1_ x B_1_b_1_) x b_2_b_2_* | $-d\left( 2p_{1}-1 \right)p_{2}$ |
| *(b_1_b_1_ x B_1_b_1_) x B_2_B_2_*  *(B_1_b_1_ x b_1_b_1_) x B_2_B_2_* | $-\frac{d\left( 4p_{1}-1 \right)\left( p_{2}-1 \right)}{2}$ |
| *(b_1_b_1_ x B_1_b_1_) x B_2_b_2_*  *(B_1_b_1_ x b_1_b_1_) x B_2_b_2_* | $-\frac{d\left( 4p_{1}-1 \right)\left( 2p_{2}-1 \right)}{4}$ |
| *(b_1_b_1_ x B_1_b_1_) x b_2_b_2_*  *(B_1_b_1_ x b_1_b_1_) x b_2_b_2_* | $-\frac{d\left( 4p_{1}-1 \right)p_{2}}{2}$ |
| *(b_1_b_1_ x b_1_b_1_) x B_2_B_2_* | $-2dp_{1}\left( p_{2}-1 \right)$ |
| *(b_1_b_1_ x b_1_b_1_) x B_2_b_2_* | $-dp_{1}\left( 2p_{2}-1 \right)$ |
| *(b_1_b_1_ x b_1_b_1_) x b_2_b_2_* | $-2dp_{1}p_{2}$ |

## Orthogonality

González-Diéguez et al. (2021) proved that all genetic effects in their GCA-model (where parental lines are completely inbred) are orthogonal assuming linkage equilibrium between QTL. For the extended models accounting for incomplete inbreeding (M2 and M3), the requirements of orthogonality are still fulfilled (Cockerham, 1954):

The mean of additive values is 0:

$$\sum_{j} z_{1j}f_{j}=0$$

$$\sum_{j} z_{2j}f_{j}=0$$

The mean of dominance deviations is 0:

$$\sum_{j} w_{j}f_{j}=0$$

where *f_j_* is frequency of parental genotype *j* in a three-way hybrid population (Table A1), and z_1j_, z_2j_ and w_j_ are as defined for M3 in Materials and Methods section (Table 2).

The mean of contrast across all possible pairs of genotypes is 0:

$$\sum_{i,j} f_{i,j}z_{1i}z_{1j}=0$$

$$\sum_{i,j} f_{i,j}z_{2i}z_{2j}=0$$

$$\sum_{i,j} f_{i,j}w_{i}w_{j}=0$$
